# Editorial: Exploring innovative therapies for rare inflammatory skin diseases

**DOI:** 10.3389/fmed.2026.1822888

**Published:** 2026-03-19

**Authors:** Dennis Niebel, Joško Miše, Joerg Wenzel

**Affiliations:** 1 Department of Dermatology, University Medical Center Regensburg, Regensburg, Germany; 2 Heinz-Werner-Seifert-Institut für Dermatopathologie KölnBonn, Bonn, Germany; 3 Department of Dermatology and Venereology, University Hospital Center Zagreb, Zagreb, Croatia; 4 Department of Dermatology, University Hospital Bonn, Bonn, Germany

**Keywords:** connective tissue disease, cutaneous, dermatomyositis, janus kinase, lichen planus, lichen sclerosus

## Introduction

Decades of translational immunological research have led to improved clinical outcomes of common dermatological conditions through the integration of mechanism-based interventions encompassing biologics and small molecules. As of 2026, there are 14 European Medicines Agency (EMA)-approved targeted treatments (i.e., blockade of molecular pathways via cytokine-/receptor-/downstream kinase-inhibition) for moderate-severe plaque psoriasis (PsO) in adults. These include TNF-α inhibitors (adalimumab, certolizumab, etanercept, and infliximab), IL17A-, IL17A/F-, and IL17-receptor inhibitors (bimekizumab, brodalumab, ixekizumab, and secukinumab), a p40/IL12/23-inhibitor (ustekinumab), p19/IL23-inhibitors (guselkumab, risankizumab, and tildrakizumab), a PDE4-inhibitor (apremilast), and a TYK2-inhibitor (deucravacitinib). In atopic dermatitis, a disease with previously no mechanism-based and long-term-appropriate therapeutics, there are now seven EMA-approved targeted treatments for moderate-severe cases in adults. These include an IL4/13-receptor inhibitor (dupilumab), IL13-inhibitors (lebrikizumab, tralokinumab) an IL31-receptor-inhibitor (nemolizumab), and JAK-inhibitors (abrocitinib, baricitinib, upadacitinib); all of the latter mentioned treatments may also be used in adolescents and some even in children. Many more innovative drugs are expected to become available in the near future with high-affinity TYK2-inhibitors (envudeucitinib, zasocitinib) and an orally available peptide directed against the IL23-receptor (icotrokinra) for PsO in the latest stage of development. However, safety remains an important concern in development of immune-modulating drugs as seen recently with rocatinlimab, a monoclonal antibody targeting the OX40-receptor. Considering occurrence of kaposi sarcomas in a low number of trial participants, the sponsor now terminated the promising phase III programs in atopic dermatitis and prurigo nodularis (1–3).

Despite the aforementioned advances, many non-communicable inflammatory skin diseases still lack licensed or even effective therapeutic options, leaving substantial gaps in patient care. *Frontiers in Medicine* dedicated a Research Topic titled “*Exploring Innovative* *Therapies for Rare Inflammatory Skin Diseases”* to shed light on recent developments in the field, welcoming submissions spanning from basic and translational to clinical research. This approach is prudent as emerging laboratory techniques, including single-cell sequencing and spatial omics, offer profound insights into inflammatory processes in individual patients, setting the stage for potential therapeutic breakthroughs. Yet, the integration of findings from basic research into clinical trials is difficult given that some rare dermatoses lack standardized diagnostic criteria, validated scores, and prognostic or predictive biomarkers. Sponsors deciding to undertake clinical trial programs in rare dermatoses may face challenges with low patient recruitment or an unbalanced patient population regarding ethnicity and sex. Even specialized centers may see only few patients in some instances. This is why case reports and case series represent valuable contributions to the literature in particular dermatoses, meaningfully propelling therapeutic standards.

Traditionally, the classification of inflammatory dermatoses relies on clinical morphology, focusing on primary and secondary skin lesions as well as distribution patterns. While morphologic features in dermoscopy and standard histopathology, reinforced by immunohistochemistry, have further refined the terminology, nowadays “omics”-approaches are used to reclassify inflammatory skin diseases (4). Eyerich & Eyerich published a landmark paper in 2018, mapping many rare dermatoses within the spectrum of four dominant immune response patterns (5). This approach is helpful to transfer targeted approaches from more common skin diseases to rarer ones (6). More recently, Seremet et al. proposed to distinguish skin diseases as molecular entities characterized by a set of dominant immune profiles (assessed via gene-expression patterns) with Th17, Th2, Th1, type-I-interferons, and myeloid modules as hallmarks of diseases (7). Later dubbed *Dermatology 2.0*., this approach may help to support diagnostic and therapeutic decision-making in ambiguous cases or patients not responding to standard treatment (8). We will introduce the articles of this collection based on this new molecular classification.

## Th1-dominant dermatoses

Lichen sclerosus (LS) is a chronic, progressive inflammatory dermatosis leading to intense itch and pain, tissue fibrosis, and an increased risk of squamous cell carcinoma. No targeted treatments are available, yet, leaving patients dependent on topical glucocorticosteroids and calcineurin inhibitors. The preliminary observational study by Rizzetto et al. explored the use of hybrid cooperative complexes of hyaluronic acid in women with vulvar LS. The treatment not only reduced hallmark findings such as hyperkeratosis and itching, but also enhanced quality of life and sexual function, suggesting meaningful restoration of vulvar tissue structure and function. This provides early evidence of a non-steroidal therapy subject to evaluation in prospective studies. Volc et al. contributed a methodical paper describing an upcoming phase IIb basket trial, which is a novel approach in immunodermatology. The trial will leverage shared mechanisms across several fibrotic skin diseases, including LS, such as early IL-13–driven fibroblast activation, to study treatment responses using both disease-specific measures and unified outcomes like the Investigator Global Assessment (IGA). Of note, treatment of LS with the pan-JAK-inhibitor delgocitinib cream will be explored in a phase III program in the near future (NCT07335588).

Alopecia areata (AA) represents another Th1-dominant disease with a variable clinical course potentially resulting in loss of all scalp hair (AA totalis) or all scalp and body hair (AA universalis). Tan et al. contributed a case report describing the trajectory of an 8-year-old boy with AA totalis carrying a novel heterozygous KRT74 variant. The child experienced rapid and sustained hair regrowth after treatment with baricitinib, highlighting the therapeutic relevance of JAK-inhibition mirrored in EMA-approvals of baricitinib and ritlecitinib as well as Food and Drug Administration (FDA)-approval of deuruxolitinib. The findings suggest that KRT74 variants may contribute to immune dysregulation in AA.

## Th2-dominant dermatoses

Within the field of type-2 inflammation, significant progress has been achieved over the last years in the care of patients suffering from dermatoses spanning from atopic dermatitis to prurigo nodularis, chronic spontaneous urticaria and bullous pemphigoid. While not particularly part of the scope of our collection, no articles were included. Readers are welcome to delve into another *Frontiers in Medicine* Research topic led by colleagues from Hannover and Seoul (https://www.frontiersin.org/research-topics/75924/type-2-inflammatory-diseases-specific-and-common-mechanisms-comorbidities-and-lessons-learned-from-targeted-therapy).

## Th17-dominant dermatoses

Palmoplantar pustulosis (PPP) is notoriously recalcitrant and represents a severe burden on quality of life for affected patients. Complex innate immune pathways including IL-36- and IL-17-activation drive the pathophysiology. Du et al. explored the potential value of the JAK1-selective inhibitor upadacitinib in a retrospective single-center study of 28 patients. Upadacitinib markedly reduced objective and patient reported outcome scores achieving rapid clinical control in most patients. While these findings may not be generalizable to non-Chinese patients, data from upcoming prospective clinical trials will help to better understand patient endotypes. While results of a phase-II trials of topical pan-JAK-inhibition are pending (NCT07013201), an upcoming phase III program will explore the safety and efficacy of bimekizumab (NCT07219420) in PPP.

Pityriasis rubra pilaris (PRP) is very rare compared to PsO causing an orange-red keratoderma, erythema with islands of spared skin and follicular hyperkeratosis. It is often quite recalcitrant, and treatment relies on drugs licensed for PsO, including conventional disease modifying antirheumatic drugs (cDMARDs) and biologics. The potential value of IL17A-inhibition is reflected in a case report by Millak et al. describing a 3-year-old girl with atypical juvenile PRP carrying a novel CARD14 variant, whose severe, treatment-refractory disease markedly improved with off-label use of ixekizumab. She achieved rapid and sustained clinical remission over almost three years without adverse effects. Zhang et al. contributed a systematic review dedicated to small-molecule drugs; 16 patients from 11 publications were identified. While publication bias may not be excluded, the results hint at meaningful clinical efficacy and good safety of apremilast and JAK-inhibitors in patients with refractory PRP, including those who previously failed cDMARDs and biologics. Larger controlled trials are needed to confirm these encouraging early results.

Hidradenitis suppurativa (HS) is a painful, chronic, and highly burdensome inflammatory skin disease. The study by Cugno et al. shows that HS patients feel significantly less involved in therapy decisions than PsO patients. Importantly, the authors demonstrate that greater involvement strongly correlates with higher satisfaction across topical, systemic, and surgical treatments, emphasizing the need for better communication and disease education in HS care. A very important emerging treatment modality is the use of GLP-1 agonists, as the incidence and severity of many dermatoses associates to obesity, including HS. Lin, Huang et al. add a case report to the existing body of evidence that GLP-1-induced weight loss may help to control inflammatory skin diseases, for instance PsO. Interestingly, the combination of GLP-1 agonism (tirzepatide) and biological treatment (ixekizumab) is already being explored in phase III clinical trials for obese patients with PsO (NCT06588283) and psoriatic arthritis (NCT06588296).

## Type I interferon-dominant dermatoses

The article by Wang, Hu et al. identifies ISG15 as a highly specific and robust diagnostic biomarker for dermatomyositis (DM), a rare inflammatory skin and muscle disease. Its integrated bioinformatics and machine-learning approach provides a modern precision-medicine framework that deepens understanding of DM pathogenesis, especially the central role of type I interferon signaling. By linking ISG15 to distinct immune-cell patterns and predicting candidate therapeutic compounds, the study opens new avenues for targeted and innovative treatments. Anti-MDA-5-positive DM is an even rarer subtype, frequently complicated by rapidly progressive interstitial lung disease that may be fatal. The article by Tokunaga et al. demonstrates that a novel multi-targeted regimen (baricitinib, rituximab, and tacrolimus) markedly improved survival, outperforming conventional cyclophosphamide-based therapy. Its integrated transcriptomic analysis provides mechanistic insight by showing that survivors experience targeted suppression of B-cell activation and interferon-driven immune pathways. Hence, the authors identified a rational, pathway-directed treatment strategy that could redefine care standards and guide future clinical trials. These two articles about DM are supplemented by an illustrative case report by Jing et al.. The article describes a patient with concurrent essential thrombocytosis driven by a JAK2-V617F mutation, in which treatment with the JAK1/2 inhibitor ruxolitinib led to rapid and marked improvement of cutaneous and muscular symptoms despite persistent thrombocytosis. By integrating a literature review of previously reported ruxolitinib-treated DM cases, the study highlights ruxolitinib's therapeutic potential in line with our own experience (9, 10).

## Myeloid-module-dominant dermatoses

Neutrophilic dermatoses represent a heterogeneous group of inflammatory skin disorders defined by neutrophil-rich infiltrates in the epidermis, dermis, or subcutis, producing diverse clinical morphologies in the absence of infection. Increasing evidence shows that many neutrophilic dermatoses share fundamental features with autoinflammatory diseases, including dysregulated innate immune responses and pathogenic activation of cytokine pathways such as IL-1 and IL-36, positioning conditions like Sweet syndrome, pyoderma gangrenosum (PG), and generalized pustular psoriasis (GPP) within a broader autoinflammatory spectrum. In our Research Topic, we included two case reports of successful treatment of GPP, a rare and potentially life-threatening condition. Li et al. describe an adult case refractory to standard treatment achieving fast flare control through spesolimab followed by long-term remission on apremilast monotherapy. In contrast, the pediatric case submitted by Wang, Dong et al. demonstrated that hydroxychloroquine may trigger GPP in children with systemic lupus erythematosus, with pustules resolving promptly after drug discontinuation and supportive therapy. Together, the reports highlight both the therapeutic potential of IL-36- and PDE4-targeted approaches and the importance of recognizing drug-induced flares to guide timely and effective management.

Sweet syndrome is an acute febrile neutrophilic dermatosis marked by painful erythematous plaques, fever, and leukocytosis, often triggered by infections, malignancy, or medications. The report of Commins et al. describes an azathioprine-induced case further complicated by a concurrent myocardial infarction underscoring the importance of prompt drug withdrawal and corticosteroid-treatment.

Palisaded neutrophilic and granulomatous dermatitis (PNGD) is a rare neutrophilic dermatosis characterized by leukocytoclastic vasculitis and palisading granulomas, often linked to systemic autoimmune or inflammatory diseases. Guo et al. provide a case report of a 6-year-old girl presenting with PNGD involving both classic extensor surfaces and atypical sites. She achieved near-complete clearance after 2 months of oral sulfasalazine, which marks the first documented successful use of this therapy for pediatric, skin-limited PNGD.

VEXAS syndrome is a severe adult-onset autoinflammatory disorder caused by somatic UBA1 mutations, leading to systemic inflammation, cytopenias, and characteristic vacuolization in myeloid precursors. In the study by Caggiano et al., a large team of researchers compared 19 male VEXAS patients with 18 male patients diagnosed with Schnitzler's syndrome and found that older age at disease onset, lymphadenopathy, anemia, and thrombocytopenia—especially in the absence of leukocytosis—strongly favored a diagnosis of VEXAS. The authors conclude that these clinical and laboratory markers can meaningfully improve diagnostic accuracy and help clinicians identify patients who should undergo UBA1 genetic testing to avoid misclassification.

## Miscellaneous

As we designed the Research Topic broadly, we also included contributions not directly linked to the wider framework of a molecular classification of inflammatory skin disease. Wang, Yang et al. provide a retrospective real-world study and found that dupilumab significantly reduced pruritus in both atopic dermatitis patients with chronic kidney disease (CKD) and those with uremic pruritus, showing similar improvements across itch severity, quality-of-life scores, and early response timepoints in both groups. Interestingly, dupilumab as well as other drugs are under investigation for chronic pruritus of unknown origin (Phase 3 trial: NCT05263206). A case report submitted by Lin, Ma et al. describes two complex skull-exposing chronic wounds that were successfully closed using a staged approach combining controlled mechanical tension with moist wound-healing therapies, demonstrating how optimizing the wound microenvironment can restart stalled repair processes. Medical databases such as FDA's Adverse Event Reporting System (FAERS) are invaluable for detecting rare or previously unrecognized adverse events because they aggregate large volumes of real-world reports, enabling signal detection that would be impossible in small clinical trials. The study by Yang et al. used two decades of FAERS data to identify 445 significant isotretinoin-associated adverse event signals, including 38 previously unlabeled events, highlighting the need for monitoring of retinoid therapy in treating inflammatory skin disease. Epidermolysis bullosa (EB) is a group of inherited blistering disorders marked by extreme skin fragility and trauma-induced blister formation due to mutations affecting structural proteins at the dermal-epidermal junction. Sun et al. report about a 59-year-old man with dystrophic EB pruriginosa carrying a COL7A1 mutation who experienced dramatic improvement in pruritus and skin lesions after 10 months of treatment with the JAK1/3 inhibitor tofacitinib.

## Summary

To summarize, our Research Topic aggregates cutting-edge articles on underserved inflammatory dermatoses, elucidating potential therapeutic advances and their translation from bench to bedside. We expect that dermatoses once considered notoriously refractory to treatment might show dramatic improvements when addressed with the right targeted approach. The full power of molecular-guided strategies backed by AI-assisted platforms is only beginning to unfold and we may anticipate therapeutic breakthroughs in underserved dermatoses in the near future.
